# Teledentistry in Pediatric Dental Care During Periods of Restricted Access: A Pilot Feasibility Study

**DOI:** 10.3390/jcm15124675

**Published:** 2026-06-16

**Authors:** Christian H. Splieth, Ahmad Al Masri, Aparna Sharma, Nithin Cordeiro, Julian Schmoeckel, Ruth M. Santamaría

**Affiliations:** 1 Department of Pediatric Dentistry, University Medicine Greifswald, 17489 Greifswald, Germany; almasria@uni-greifswald.de (A.A.M.); sharma3aparna@outlook.com (A.S.); nithin.cordeiro@uni-greifswald.de (N.C.); julian.schmoeckel@uni-greifswald.de (J.S.); ruth.santamaria@uni-greifswald.de (R.M.S.); 2 Department of Orthodontics, University Medicine Greifswald, 17489 Greifswald, Germany

**Keywords:** teledentistry, pediatric emergency services, children, screening, triage

## Abstract

**Background**: For maintaining specialized dental treatment all year and in remote areas, teledentistry might offer a valuable approach. **Aim**: To prospectively pilot the feasibility and practicality of pediatric teledentistry during a period of restricted access during the Christmas break 2025/2026. **Methods**: The organizational and clinical parameters of emergency patients during the Christmas break 2025/2026 were registered for a prospective analysis. Descriptive statistics focused on the feasibility of teledentistry with categorizing treatment as only counseling without further dental contact, postponing to later regular clinic times, immediate acute pain management in-person. **Results**: Of the twenty-four registered patient contacts (eight female, 33%; age 1–17 years, mean 8.0 ± 3.5), 25% presented with only primary dentition and 62% of the problems addressed primary teeth. Seventy-one percent reported pain as chief complaint, leading to the diagnosis of abscess (21%), normal exfoliation or a remaining root and trauma (17% each), and simple carious defects or teeth without relevant dental pathology (13% each). These diagnoses required a timely emergency treatment in presence (46%), while the other patients could be treated with primary teledentistry consultations only (29%), while 13% received antibiotics initially and later regular treatment and in 13% the in-person diagnostics revealed no relevant dental pathology (13%). The clinical emergency treatment consisted of extractions (17%), fillings (13%) or endodontic treatment (13%). Further treatment need remained for extractions (29%) or treatment under sedation/general anesthesia (8%). **Conclusions**: Teledentistry was feasible for initial screening or triage during the holiday season. One third of the cases could be reduced to teledentistry as consultation only, leaving a relevant need for emergency services and also follow-up clinical visit in presence at regular practice times.

## 1. Introduction

Oral diseases affect more than 3.5 billion people worldwide, highlighting the urgent need to improve access to and affordability of dental care [1]. In this context, information and communication technologies, including teledentistry, have emerged as promising tools for improving the accessibility of healthcare, with their relevance becoming particularly evident during the COVID-19 global pandemic [2,3]. In addition, oral diseases are highly prevalent and socially determined, disproportionately affecting individuals from lower socioeconomic backgrounds who have limited access to preventive and dental care services. Socioeconomic status affects access to material resources, such as oral hygiene products and professional care, as well as non-material factors, including health literacy and access to healthcare. Even in highly developed healthcare systems, access to preventive services is not equally distributed, with individuals of a higher socioeconomic status benefiting more from the available resources. Consequently, disparities in access to care remain a primary cause of inequalities in oral diseases [4].

Teledentistry was originated in the late 20th century as part of telemedicine and was initially developed to overcome geographical barriers in remote and underserved regions [5,6]. Early applications demonstrated the feasibility of remote diagnosis through digital data exchange [6,7]. Since then, advances in imaging technologies, electronic health records and internet connectivity have enabled its expansion into public health, specialist consultations and patient management [8,9,10,11,12]. There is increasing evidence to support its reliability in screening, diagnosis, triage and follow-up care, as well as its potential to improve patient engagement and reduce inequalities in oral health, particularly when integrated with artificial intelligence [7,12,13,14].

In addition, teledentistry has shown potential to improve the efficiency of healthcare systems by reducing unnecessary clinical visits and optimizing the allocation of resources, thereby contributing to more cost-effective care delivery [2]. However, challenges remain, including concerns relating to data protection, variability and validity in diagnostic accuracy, and the need for standardized protocols and options for implementation in clinical practice.

Importantly, the relevance of teledentistry is no longer limited to geographically isolated settings. In Europe, ongoing demographic aging and associated workforce retirements are expected to intensify shortages in dental care, posing significant challenges for maintaining continuous and equitable service provision, especially in less populated areas and during periods of reduced service availability. Despite the historically strong performance of healthcare systems, these developments necessitate innovative and flexible approaches. In this context, teledentistry, particularly as a triage and supportive tool, offers a valuable strategy to sustain care delivery while reducing the burden on conventional in-office services [14,15].

In pediatric populations, barriers to accessing dental care are particularly pronounced, as children depend on caregivers and organized healthcare systems, which can make timely preventive and diagnostic interventions more challenging [16]. The early detection and management of oral diseases is crucial to prevent long-term consequences, which highlights the need for accessible and flexible care models.

Although in oral health existing evidence syntheses have examined specific aspects of teledentistry, such as its application in dental education, the recognition of particular oral conditions, and its use in orthodontics for patient screening and support [2,8,12,14,17,18,19], comprehensive evaluations of its real-world impact on access to care, service delivery, practicality and clinical feasibility remain limited. In particular, despite growing recognition of the potential role of teledentistry in pediatric dental care, there is a lack of real-world studies evaluating its practical implementation during short-term disruptions to dental services, such as national holidays or other temporary service disruptions. Therefore, the present pilot study aimed to assess the feasibility and practicality of teledentistry in pediatric dentistry during periods of limited access to care, particularly during the Christmas holiday period.

## 2. Materials and Methods

For the two-week Christmas break from Monday 22 December 2025 to Friday 2 January 2026, most private dental clinics close completely. Theoretically, the dental association provides an emergency dental service, but this is restricted to 6 to 8 pm at often distant private dentists (30 to 50 km drive). In addition, the emergency services in the University Department of Facial Maxillary Surgery are continuously available, but only for severe cases, especially facial trauma or dental abscess with a tendency to spread (risk of sepsis). In these two weeks, the Department of Pediatric Dentistry at the University of Greifswald was available only for emergency services. During other times of the year, more dental services are continuously available due to alternating closure of practices for vacation. This gives an excellent opportunity during the Christmas break to collect an almost complete patient sample for assessing the feasibility of teledentistry.

This exploratory pilot study considered the feasibility of implementing and using a teledentistry service within routine pediatric dental care in pragmatic terms. Specifically, we assessed the service’s feasibility by looking at how many people used it, whether presenting conditions could be assessed remotely, whether it supported clinical decision-making and triage, and whether it directed patients to immediate or deferred care appropriately. There was no predefined feasibility framework or set of specific feasibility outcomes. A team of specialized pediatric dentists was assigned according to a rotation plan to cover all working days in these two weeks, while the dental clinic was completely closed on weekends and national holidays (24–28 December, 1 January).

The patient flow followed a standardized protocol:

1. Whenever the department was contacted for a pediatric dental emergency treatment, a dental nurse questioned the parents/guardians about the emergency case and consulted the dentist about the situation.
2. Clear cases of emergency like trauma were treated directly without teledentistry.
3. Otherwise, teledentistry was used, with the dentist asking about the medical and dental history as well as about the acute situation to decide if teledentistry was sufficient, a prescription at a pharmacy or a visit in the clinic was needed immediately or later.
4. If teledentistry was performed, the parents/guardians were given a Zoom link along with the information on data protection issues and asked to log in with their smartphone. The dentist was in charge of the Zoom link and hosting the connection. In face to face contact the parents/guardians were requested to provide an image of the child, their face and, if possible, the affected tooth, which was sent per email for further assessments. These transfers were done via the data-safe programs used by the Medical University of Greifswald to ensure confidentiality.
5. Using the information collected through the parents/guardians, the clinical pictures and the x-rays, if available for in-house patients, the dentist reached a primary diagnosis and decided accordingly for either an urgent need to see and treat the patient clinically or advice for the management of the case until regular clinical treatments were possible (for example prescription of antibiotics).

All treatments were regularly documented thoroughly in a specialized dental computer program (IVORIS^®^; Falkenstein, Germany). A retrospective analysis of the data from the digital patient’s records was performed to assess the feasibility of teledentistry in dental emergencies. All patients who contacted the clinic were included in this study without any restrictions. The patient and treatment data were completed into the Excel sheet (Microsoft Corp. 365) and transferred into a SPSS data bank (IBM Corp.; version 30.0; New York, NY, USA). One trained researcher was responsible for checking the patients’ digital records from the above-mentioned time period for plausibility retrospectively and completing the data in the Excel sheet, especially regarding the final treatment and feasibility of the feasibility for teledentistry. An experienced senior researcher double-checked the data, especially in uncertainties in the data collection procedure.

The collected data included:

- Date, weekday, and time of patient contact.
- Age and gender of the patient.
- Registration status in the clinic (new/referred vs. known patients).
- Chief complaint (pain, abscess or fistula, dental trauma, loss of restoration, loss of tooth) and systemic involvement (all dichotomized as yes/no).
- Tooth number and type (primary or permanent).
- Diagnostic procedures (medical history, clinical examination if performed, x-ray availability).
- Main diagnosis (exfoliation of primary tooth, remaining root, caries media without painful pulp involvement, pulpitis with pain, pulp necrosis with negative vitality, abscess, trauma).
- Treatment provided (only counseling, restoration, endodontics, extraction, need for future sedation or general anesthesia [GA], or other treatments).
- Type of management for the feasibility of teledentistry (only counseling without further dental contact on this issue, postpone to later treatment at regular clinic times after holiday break, immediate, acute pain management in-person with referral to final treatment at regular clinic times after holiday break, immediate definitive treatment in-person without further dental contact on this issue).

### Statistical Analysis (Descriptive)

After a primary descriptive analysis (frequencies, range, mean/median), some data like time of patient contact, were recategorized (e.g., early and late morning or afternoon) and re-evaluated. In addition, patterns in chief complaints, diagnoses, and treatment measures were analyzed for primary and permanent dentition.

Finally, patients were grouped into categories based on management pathway: the feasibility of teledental contact only, the need of immediate in-person dental treatment, or the possibility of delayed or follow-up treatment within regular practice hours.

An ethical approval for the anonymous retrospective analysis of patient’s digital records for research and quality management purposes is available from the ethical commission of the University of Greifswald (Protocol Number BB28/16).

## 3. Results

The twenty-four registered patient contacts (eight female, 33%; 16 male, 66%; Table 1) had an age range from 1 to 17 years old (mean 8.0 ± 3.5 years). The age frequencies showed an almost perfect normal distribution with 7–9 years of age being the strongest groups (17% each); 25% of the children had only primary dentition (<6 years) and only 8% presenting with a permanent dentition, while the rest had mixed dentition. An overview of the general characteristics of the included patients and the collected data is shown in Table 1.

Most patients came directly after the weekend on Monday (54%, Table 1) and already early in the morning (33%) or the late morning (29%). About one third were external/new patients, who have not been registered at the dental clinic of the University as patients (37%).

**Figure 1:**
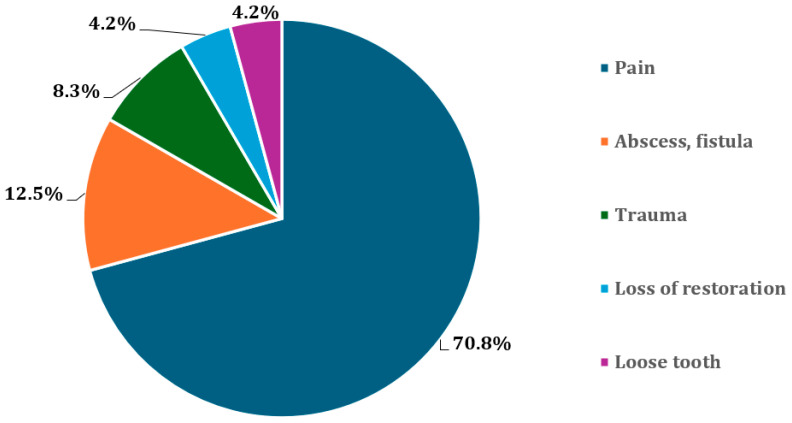
Chief complaint of emergency patients during Christmas breaks (*n* = 24).

The chief complaint and main reason for the consultation given by the patients or their parents was toothache (71%, Figure 1), followed by abscess or fistula (13%), while dental trauma was mentioned rarely (8%). The patient mainly presented only with dental problems (88%) and, therefore, without systemic involvement (22%).

Most affected teeth were in the primary dentition (62%) and an almost equal frequency in the maxilla and mandible.

For most of the patients, an x-ray was not needed or not feasible due to low cooperation (71%).

The most frequent diagnosis was abscess (21%, Table 2), followed by normal exfoliation or a remaining root and trauma (each 17%). Simple carious defects or teeth without relevant dental pathology like regular exfoliation were diagnosed rarely (13% each).

For finalizing a treatment plan, not only the diagnosis (Table 2) has to be taken into account, but also the feasibility of the cooperation of the child or the availability of general anesthesia in case of limited cooperation. Thus, given these diagnoses a direct emergency treatment by the dentist in presence was indicated and feasible (46%), while the other patients could be treated with teledentistry consultations only (29%, Table 1). For 13% antibiotics had to be prescribed initially with regular treatment at later appointments and for another 13% the essential diagnostic measures revealed such as x-rays revealed no relevant dental pathology, e.g., normal exfoliation process or black stain.

**Figure 2:**
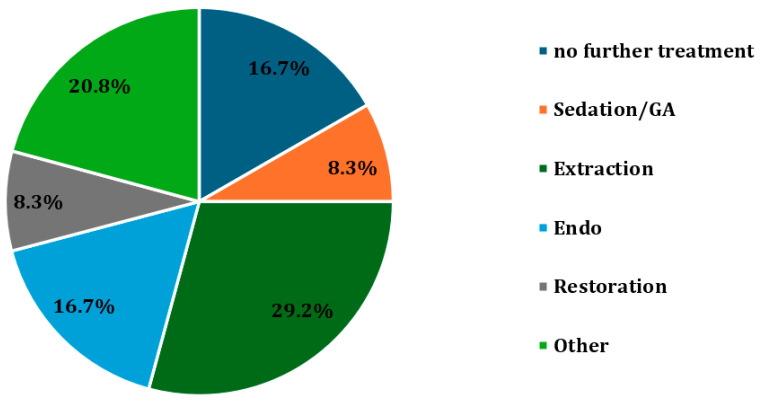
Final treatment needed after emergency visit during Christmas breaks (*n* = 24).

The clinical emergency treatment range included a variety of different measures such as extractions (17%, Figure 2), fillings (13%) or endodontic treatment such as pulpectomy/root canal treatment (13%), trepanation (8%) or pulpotomy (4%). After this emergency treatment further treatment need remained for extractions (29%, Figure 2), endodontic measures (17%), restorations or treatment under sedation or general anesthesia (8%). This further treatment was mostly planned at a specialized pediatric clinic (54%) and to a lesser extent at the local general dentist (38%). In summary, one third of the cases could be reduced to teledentistry as consultation only during the holiday season, but partly also requiring a follow-up clinical visit in person at regular practice times.

## 4. Discussion

This study explored the feasibility of teledentistry in a specialist pediatric dental university setting, where patients are often referred from a large geographical area. As a regional referral center, this clinic manages children with complex dental conditions, frequently requiring families to travel considerable distances. Although many families are highly motivated to attend in-person visits, access can still be challenging due to travel burden, the young age of patients, and the logistical demands of childcare [6]. These barriers suggest the potential value of complementary care models that can facilitate access to specialist advice.

The findings of the present study show that remote consultations are particularly useful for initial assessment and clinical prioritization. The clustering of contacts following weekends may indicate that families use teledentistry as an accessible first point of contact when concerns arise outside regular service hours. The spectrum of presentations ranged from cases without relevant acute pathology and physiological processes such as normal exfoliation to acute conditions including abscesses and dental trauma. While certain diagnoses, such as retained roots, require radiographic confirmation, and conditions like dental trauma necessitate in-person management, these could still be identified early and appropriately prioritized through remote contact. In this cohort, 46% of patients required immediate clinical intervention, whereas approximately one third could be managed through consultation alone or treatment could be safely postponed. While this finding highlights that many pediatric dental emergencies ultimately require face-to-face assessment and treatment, it also demonstrates the value of teledentistry as an effective triage and prioritization tool. This distribution is similar to results from other specialized centers [20,21], where about one third of the patients received no immediate treatment except for the prescription of antibiotics, but a high proportion needed extractions or pulp treatment. This indicates that in the present cohort, a substantial proportion of cases could be initially handled without direct clinical contact, while still ensuring that urgent conditions were identified and treated in a timely manner. In particular, abscesses, the most frequent diagnosis, could be recognized remotely and referred for urgent medical care when necessary. These findings suggest that teledentistry may serve as an important complement to conventional care, particularly during periods of restricted access to dental services.

The majority of cases were initially assessed without radiographs (71%), often due to teledentistry or simply limited patient cooperation. This reflects the realities of a pediatric setting and highlights that decision-making in many situations relies primarily on history and visual assessment. Within this context, remote consultation supports structured triage and early clinical decision-making, including the indication for pharmacological management such as analgesics or antibiotics when appropriate.

In pediatric dental care, remote consultation may also reduce the burden of unnecessary or stressful initial visits, particularly for very young children. It allows parents to receive timely guidance, reassurance, and clear recommendations regarding the urgency of further care. Personalized remote communication may also support caregiver understanding and engagement in care pathways. Similarly to previous reports suggesting the diagnostic utility of teledentistry [12,18], the present study indicates that remote consultation may be a useful tool for initial assessment, prioritization, and triage in specialized pediatric dental settings. These findings suggest that teledentistry may be considered as a complementary triage tool for the initial assessment of pediatric dental emergencies, before proceeding to further systematic management protocols. This should take into account the urgency of the treatment, as well as the applicability of clinical management, considering factors such as the child’s level of cooperation, and the time slots available in the clinic [21].

The effective implementation of teledentistry in pediatric dental care extends beyond the technical feasibility of remote consultations and depends on caregiver-related factors, including oral health literacy, digital competence, and access to appropriate technology [19]. Parents and caregivers play a central role in recognizing symptoms, providing accurate clinical information, transmitting photographs or videos when required, and understanding the urgency of recommended actions. Limitations in any of these areas may affect the feasibility and effectiveness of teledentistry-based care [19,21]. Future research should therefore explore the extent to which oral health literacy and digital competence affect equitable access to, and successful use of, teledentistry in pediatric dental care. This study has several limitations that should be considered when interpreting the findings. As a pilot study with a modest sample size conducted in a single specialist pediatric dental center, the generalizability and extrapolation of the findings is limited. As a result, the observed utilization and perceived value of teledentistry may differ from settings in which emergency dental services remain more readily accessible. Nevertheless, the study provides insight into the potential role of teledentistry as a triage and support tool during periods of restricted service availability, and further research is required to evaluate its effectiveness under different service configurations. On the other hand, a strength of the study is its real-world clinical setting in a specialist pediatric referral center, reflecting routine clinical practice and the practical challenges of delivering urgent pediatric dental care during periods of restricted access. Additionally, the retrospective nature of the data collection may introduce a risk of information bias, as the analysis relied on existing clinical documentation. However, clinical documentation and data entry in our department follow standardized procedures performed by trained staff independently of this study, including structured preparation for periods of restricted service provision, which may have helped to reduce inconsistencies in data recording. Nevertheless, potential bias related to data extraction and interpretation cannot be entirely excluded. Furthermore, the study was designed to assess feasibility and descriptive clinical utility rather than diagnostic accuracy or comparative effectiveness. Therefore, the findings should be interpreted as exploratory and hypothesis-generating.

In conclusion, this pilot study suggests that teledentistry can be feasibly integrated into pediatric dental care as a tool for early assessment, triage, and patient guidance. Its use is practicable and may be particularly helpful not only in pandemic situations such as the COVID-19 [3,22,23,24,25], but also in situations where the access to dental care is limited as in Christmas break as in this study. Although not directly assessed in the present study, teledentistry may have the potential to reduce unnecessary travel, and support more sustainable healthcare delivery. These potential benefits should be explored in future research. Its primary role appears to be to support clinical decision-making and facilitates access to appropriate care, while recognizing the continued need for in-person assessment and treatment where required. Given the pilot nature, single-center setting, and limited sample size of this study, these findings should be interpreted cautiously and confirmed in larger prospective studies.

## Figures and Tables

**Table 1:** Characteristics of emergency patients during Christmas break (*n* = 24).

Variable		Frequency (*n*)	Percentage (%)
Day of the emergency	Monday	13	54.2%
	Tuesday	9	37.5%
	Friday	2	8.3%
Time	Early morning	8	33.3%
	Late morning	7	29.2%
	Early afternoon	7	29.2%
	Late afternoon	2	8.3%
Age	1–5 years	5	20.8%
	6–11 years	16	66,7%
	12–17 years	3	12.5%
Gender	Female	8	33.3%
	Male	16	66.7%
new/referred vs. known patients	new/referred	9	37.5%
	Known	15	62.5%
Systemic Involvement	No	21	87.5%
	Yes	3	12.5%
Affected Jaw	Maxilla	13	54.2%
	Mandible	11	45.8%
Type of affected tooth	Primary	14	58.3%
	Permanent	10	41.7%
Diagnosis x-ray	No x-ray	17	70.8%
	Caries Media	5	20.8%
	Pulp involvement	1	4.2%
	Abscess	1	4.2%
	Remaining root	1	4.2%
Prescription of Antibiotics	No	21	87.5%
	Yes	3	12.5%
Clinical treatment performed	No	13	54.2%
	Yes	11	45.8%
Type of management	Counseling only	7	29.2%
	Postpone to regular appointment	5	20.8%
	Acute pain management	6	25.0%
	Definitive treatment	6	25.0%

**Table 2:** Main diagnoses of emergency patients during Christmas breaks (*n* = 24).

Diagnosis	Frequency (*n*)	Percentage (%)
Healthy/no relevant pathology	3	12.5%
Exfoliation/remaining root	4	16.7%
Caries Media	3	12.5%
Pulpitis	3	12.5%
Pulp necrosis	2	8.3%
Abscess or Fistula	5	20.8%
Trauma	4	16.7%

## Data Availability

Due to data protection regulations stipulated in §§37–37d of the Landeskrankenhausgesetz Mecklenburg-Vorpommern (LKHG M-V), the dataset generated and analyzed during the current study cannot be made publicly accessible. However, data may be provided by the corresponding author upon reasonable request, subject to approval by the institutional ethics committee of University Medicine Greifswald and the responsible data protection authority.
